# Cohort Profile: Guangzhou Nutrition and Health Study (GNHS): A Population-based Multi-omics Study

**DOI:** 10.2188/jea.JE20230108

**Published:** 2024-06-05

**Authors:** Chu-Wen Ling, Haili Zhong, Fang-fang Zeng, Gengdong Chen, Yuanqing Fu, Cheng Wang, Zhe-Qing Zhang, Wen-Ting Cao, Ting-Yu Sun, Ding Ding, Yan-Hua Liu, Hong-Li Dong, Li-Peng Jing, Wenhua Ling, Ju-Sheng Zheng, Yu-Ming Chen

**Affiliations:** 1 Department of Epidemiology, Guangdong Provincial Key Laboratory of Food, Nutrition and Health, School of Public Health, Sun Yat-Sen University, Guangzhou, China; 2 Department of Epidemiology, School of Medicine, Jinan University, Guangzhou, China; 3 Department of Obstetrics, Foshan Institute of Fetal Medicine, Southern Medical University Affiliated Maternal & Child Health Hospital of Foshan, Foshan, China; 4 Key Laboratory of Growth Regulation and Translational Research of Zhejiang Province, School of Life Sciences, Westlake University, Hangzhou, China; 5 Department of Clinical Nutrition, Sun Yat-sen Memorial Hospital, Guangzhou, China; 6 Department of Nutrition and Food Hygiene, Guangdong Provincial Key Laboratory of Tropical Disease Research, School of Public Health, Southern Medical University, Guangzhou, China; 7 International School of Public Health and One Health, Hainan Medical University, Haikou, China; 8 Global Health Research Center, Guangdong Provincial People’s Hospital, Guangdong Academy of Medical Sciences, Guangzhou, China; 9 Department of Nutrition, the First Affiliated Hospital of Zhengzhou University, Zhengzhou, Henan, China; 10 Scientific Education Section and Department of Child Healthcare, Affiliated Maternity & Child Health Care Hospital of Nantong University, Nantong, China; 11 Department of Epidemiology, School of Public Health, Lanzhou University, Lanzhou, China; 12 Department of Nutrition, School of Public Health, Sun Yat-sen University, Guangzhou, China

**Keywords:** metabolic health, population-based, cohort study, nutrition, multi-omics

## Abstract

**Background:**

The Guangzhou Nutrition and Health Study (GNHS) aims to assess the determinants of metabolic disease in nutritional aspects, as well as other environmental and genetic factors, and explore possible biomarkers and mechanisms with multi-omics integration.

**Methods:**

The population-based sample of adults in Guangzhou, China (baseline: 40–83 years old; *n* = 5,118) was followed up about every 3 years. All are tracked via on-site follow-up and health information systems. We assessed detailed information on lifestyle factors, physical activities, dietary assessments, psychological health, cognitive function, body measurements, and muscle function. Instrument tests included dual-energy X-ray absorptiometry scanning, carotid artery and liver ultrasonography evaluations, vascular endothelial function evaluation, upper-abdomen and brain magnetic resonance imaging, and 14-day real-time continuous glucose monitoring tests. We also measured multi-omics, including host genome-wide genotyping, serum metabolome and proteome, gut microbiome (16S rRNA sequencing, metagenome, and internal transcribed spacer 2 sequencing), and fecal metabolome and proteome.

**Results:**

The baseline surveys were conducted from 2008 to 2015. Now, we have completed 3 waves. The 3rd and 4th follow-ups have started but have yet to end. A total of 5,118 participants aged 40–83 took part in the study. The median age at baseline was approximately 59.0 years and the proportion of female participants was about 69.4%. Among all the participants, 3,628 (71%) completed at least one on-site follow-up, with a median duration of 9.48 years.

**Conclusion:**

The cohort will provide data that will be influential in establishing the role of nutrition in metabolic diseases with multi-omics.

## INTRODUCTION

The increase in the prevalence of chronic diseases is one of the biggest challenges for the healthcare system. Cardiovascular diseases (CVDs) are the leading cause of death globally, taking an estimated 17.9 million lives each year.^1^ Between 2000 and 2040, the ageing of the Chinese population alone is predicted to cause a 200% increase in deaths from CVDs.

Multi-omics data is an excellent resource for discovering novel biomarkers of cardiometabolic disease outcomes. In addition, omics analyses can provide valuable insights into the mechanism of chronic diseases, which will promote the development of effective personalized therapy that leads to clinical care tailored to the individual patient. Finally, we planned to integrate exposure, multi-omics biological profiles, and clinical data to obtain a multidimensional biomarker to identify the individual risk and possible treatment for chronic diseases.

The prospective Guangzhou Nutrition and Health Study (GNHS) project was established to assess the determinants of risk of common chronic diseases (mainly metabolic diseases) and changes in their relevant indices in nutritional aspects, as well as other environmental and genetic factors, and explore possible mechanisms with multi-omics integration. In this cohort, the original GNHS and another cohort study (the controls of a case-control study of hip fractures [CCFH]) have been integrated into the one GNHS project. The GNHS baseline study conducted in China is the baseline study of the original GNHS (2008–2013) and the CCFH (2009–2015). The GNHS project was designed as joint research, in which the same research group jointly managed the sub-cohorts with the same aim.

## METHODS

### Study participants

The GNHS project is a community-based prospective cohort study. The participants in the original GNHS were recruited from multiple communities covering the majority of Guangzhou city via the following measures: local advertisements, health talks, and referrals. The baseline study includes about 4,048 healthy Chinese adults living in Guangzhou city (South China) for >5 years, aged 40–80 years, and recruited between 2008 and 2013.

The participants in the CCFH baseline (52–83 years old) were recruited in Guangzhou City, Guangdong Province, China, from June 2009 to August 2015. A total of 887 healthy residents in communities in Guangdong Province and 183 patients who had been hospitalized within a week with one of the following diseases were included: pneumonia, benign ophthalmic, influenza, otorhinolaryngologic tumor, cataract in one eye or acute surgical diseases, which were not the outcomes on which GNHS focused.

The study protocol of the GNHS project was approved by the Ethics Committee of the School of Public Health at Sun Yat-sen University (2018048) and was performed following the principles outlined in the Declaration of Helsinki. Written informed consent was received from all participants prior to the start of the investigation.

After completing the baseline examination, a total of 5,118 participants were recruited during 2008–2015 in the GNHS project. Table 1 shows characteristics information by sub-cohorts at baseline; 4,048 participants (4,048/5,118; 79.1%) and 1,070 participants in the CCFH (1,070/5,118; 20.9%) contributed to the GNHS project. Overall, the median age at baseline was approximately 59.0 years; the proportion of female participants was about 69.4% (3,550/5,118); the average percentage of the secondary high school stood at 41.6% (2,130/5,118). Compared to the CCFH participants at baseline (2008–2015), the original GNHS participants (2008–2013) had a similar distribution of body mass index and alcohol drinkers, less physical activity, and more energy intake; were more likely to be younger, men, higher educated, married or living together with others, smokers, and tea drinkers; and had a lower proportion of calcium supplement users, multivitamin users, and household income ≥3,001 yuan/month/person (Table 1). Baseline characteristics differed between women and men in the GNHS project (Table 1).

**Table 1.** Baseline characteristics^a^ (*n* = 5,118)

	By sub-cohorts			By sex		
	
	CCFH	GNHS	*P*-value	Women	Men	*P*-value
*n* (%)	1,070 (20.9)	4,048 (79.1)		3,550 (69.4)	1,562 (30.6)	

	Median (IQR) or *n* (%)					
**Age, years**	72.0 (66.0–76.0)	58.0 (54.0–62.0)	**<0.001**	58.6 (54.0–66.0)	61.0 (56.0–67.3)	**<0.001**
**Gender**			**<0.001**	—	—	—
Women	794 (74.2)	2,756 (68.1)				
Men	276 (25.8)	1,286 (31.8)				
Missing/unclassified	0 (0.0)	6 (0.1)				
**BMI, kg/m^2^**	23.2 (21.5–24.9)	23.2 (21.2–25.3)	0.690	23.0 (21.1–25.0)	23.7 (21.8–25.5)	**<0.001**
**Education**			**<0.001**			**<0.001**
Junior high school or below	509 (47.6)	1,223 (30.2)		1,270 (35.8)	462 (29.6)	
Secondary high school	353 (33.0)	1,777 (43.9)		1,554 (43.8)	576 (36.9)	
College degree or above	207 (19.3)	991 (24.5)		696 (19.6)	505 (32.1)	
**Household income, ** **Yuan/month/person**			**<0.001**			**<0.001**
⩽ 500	15 (1.4)	86 (2.1)		59 (1.7)	40 (2.7)	
501–2,000	125 (11.7)	1,067 (26.4)		888 (25.0)	304 (19.5)	
2,001–3,000	273 (25.5)	1,774 (43.8)		1,445 (40.7)	602 (38.5)	
⩾ 3,001	400 (37.4)	1,075 (26.6)		963 (27.1)	512 (32.8)	
**Marital status**			**<0.001**			**<0.001**
Married or living together	791 (73.9)	3,622 (89.5)		2,918 (82.2)	1,495 (95.7)	
Divorce/Separation/Widowed	273 (25.5)	366 (9.0)		588 (16.6)	51 (3.3)	
Unmarried/Unclassified	6 (0.6)	60 (1.5)		44 (1.2)	16 (1.0)	
**Smokers** ^b^	135 (12.6)	674 (16.7)	**<0.001**	33 (0.9)	776 (49.7)	**<0.001**
**Alcohol drinkers** ^c^	77 (7.2)	280 (6.9)	0.100	100 (2.8)	257 (16.5)	**<0.001**
**Tea drinkers** ^d^	540 (50.5)	2,119 (52.3)	**0.023**	1,576 (44.4)	1,083 (69.3)	**<0.001**
**Physical activity, MET-h/d** ^e^	37.2 (28.3–47.4)	35.5 (30.4–48.8)	**0.013**	36.3 (30.4–48.6)	34.9 (29.7–46.7)	**<0.001**
**Energy intake, kcal/d**	1,506.3 (1,288.4–1,797.2)	1,681.0 (1,404.3–2,064.0)	**<0.001**	1,587.8 (1,318.9–1,911.0)	1,801.8 (1,492.7–2,183.1)	**<0.001**
**Calcium supplement users**	455 (42.5)	1,261 (31.2)	**<0.001**	1,383 (39.0)	333 (21.3)	**<0.001**
**Multivitamin supplement users**	307 (28.7)	855 (21.1)	**<0.001**	893 (25.2)	269 (17.2)	**<0.001**

BMI, body mass index; CCFH, the controls of a case-control study of hip fractures; GNHS, Guangzhou Nutrition and Health Study; IQR, interquartile range; MET, metabolic equivalent.

^a^Continuous variables were described as medians ± quartile range in non-normal distribution, assessed by Wilcoxon tests; categorical variables were described with numbers (%), assessed through chi-square tests.

^b^Smokers were defined as having smoked at least 1 cigarette every day for at least 6 consecutive months.

^c^Alcohol drinkers were defined as taking an alcoholic drink at least once per week for at least 6 consecutive months at any time.

^d^Tea drinkers were defined as taking at least 1 cup of tea per week in the previous 6 months.

^e^Physical activity included daily household chores, walking, standing, stair-climbing, bike-riding, hard physical labor, moderate physical labor, and mild physical labor, assessed by metabolic equivalent (MET) hours per day.

### Follow-up procedure

**Figure 1.**
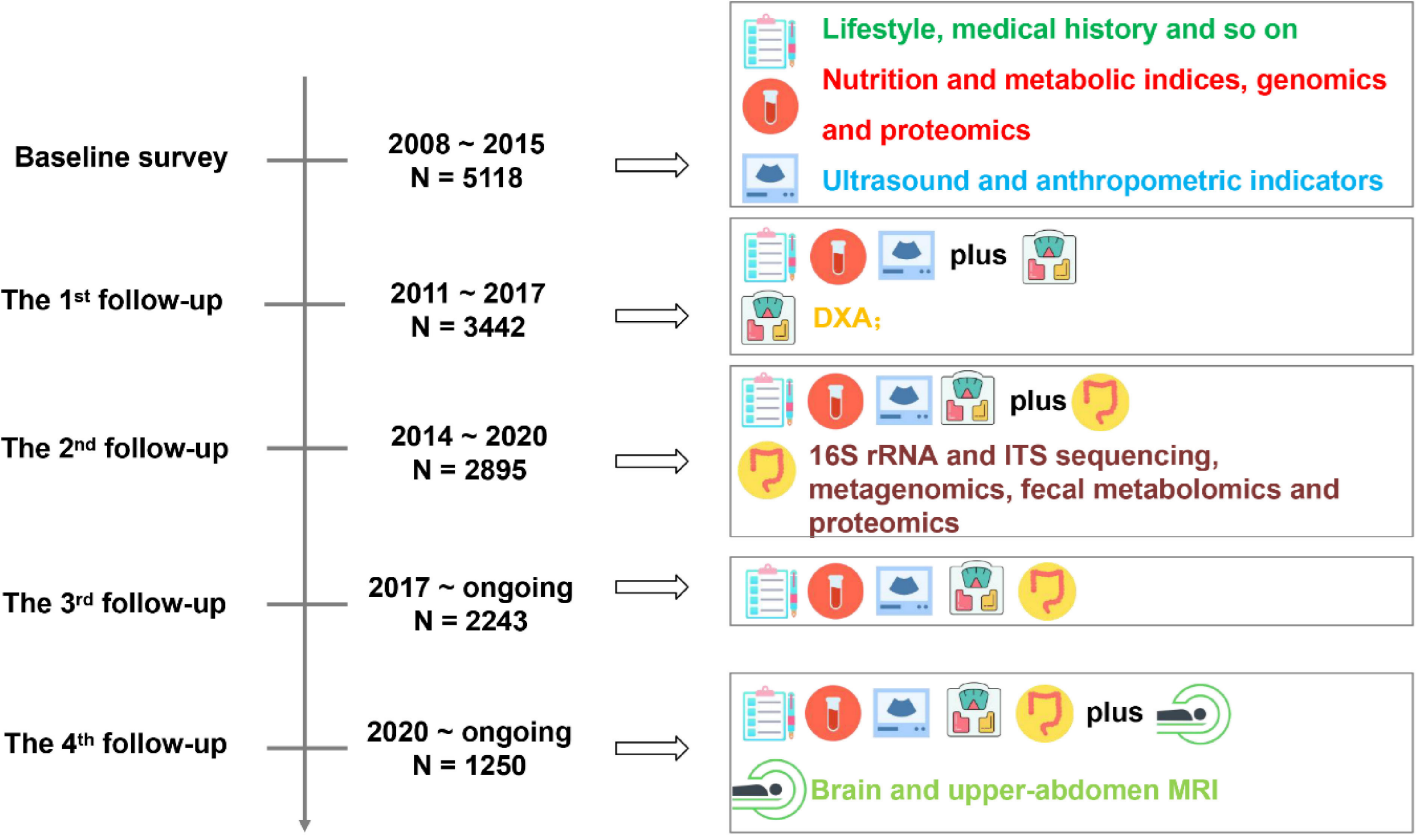
The research project of Guangzhou Nutrition and Health Study (GNHS). DXA, Dual-energy X-ray absorptiometry; ITS, internal transcribed spacer; MRI, magnetic resonance imaging.

Figure 1 shows the primary data items collected approximately every 3 years from 2008. Among the population-based samples (GNHS Baseline study: 2008–2015, *N* = 5,118), 3,628 (71%) completed at least one on-site follow-up, with a median duration of 9.48 years. All will be tracked via health information systems. eTable 1 shows that participants who lost at the 2nd follow-up had more risk factors for chronic diseases (for example, older age, lower level of education, a lower proportion of physical activity and marriage, and a higher proportion of smokers and alcoholic drinkers).

### Data measurement

In the baseline and follow-up phases, all participants were invited to Sun Yat-sen University by telephone every 3 years for face-to-face interviews, specimen collections, and body examinations. The main contents of the field works were similar at each visit. In addition, blood, urine, fecal, and saliva samples were collected as soon as possible. eTable 2 describes a broad overview of the data collected so far. The collected data included the following major sections: general information and lifestyle factors, health and well-being, physical examinations, instrumental examinations, laboratory tests, and multi-omics data.

### General information and lifestyle

In this part, face-to-face interviews using structured questionnaires were conducted to collect the following information: demographic and socioeconomic characteristics, employment, social support and participation, health-related behaviours,^2^ physical activities, and dietary assessments. Dietary assessments used a 79-item Food Frequency Questionnaire (FFQ) or 7-day image-based food diary. Changes in eating habits and using dietary supplements were also assessed. The FFQ questionnaire included 79 items,^3^ consisting of eight categories (staples, beans and bean products, vegetables, fruits, food of animal origin, nuts, beverages, and soups).

### Health and well-being

This section is described in detail in eMaterial 1.

### Physical examinations

We conducted the following physical examinations: anthropometric measurements (eg, body weight and height, waist, hip, neck, and calf circumferences), muscle function (eg, handgrip strength, step speed, Chair Stand Testing, and balance function), blood pressure, and 14-day bracelet motion monitoring.

### Instrumental examinations

Participants received extensive instrumental examinations as part of their participation in the GNHS project. The assessment included Dual-energy X-ray absorptiometry (DXA) scanning,^4^ ultrasonography evaluations,^5^ vascular endothelial function evaluation, cardiopulmonary exercise testing, upper-abdomen and brain magnetic resonance imaging (MRI), and 14-day real-time continuous glucose monitoring tests. DXA scanning is used to measure bone mineral density (BMD)^4^ and bone mineral content (BMC) at the whole body, lumbar spine segment 1–4 (spine L1–L4) and left hip, fat^6^ and lean mass^7^ at the whole body and sub-regions, and bone geometry information at the left hip. Ultrasonography was used to evaluate carotid artery intima-media thickness,^5^ plaque, stiffness, and the degree of fatty liver.^8^ The brain MRI was used to study brain tissue’s microstructure and investigate brain function without requiring the subject to undertake a specific task. In addition, we conducted upper-abdomen MRI to assess the structure and contents of fat and iron of the liver, fat and muscle mass, and vertebral bone marrow fat, and to help identify renal angiomyolipoma and malignant renal tumors. A 14-day continuous glucose monitoring was used to determine glycemic responses to various usual daily foods (by a 7-d image-based food diary) using three-type standard breakfast as internal calibrators and physical activities assessed by 14-day bracelet motion monitoring.

### Laboratory tests

In all examination waves, a broad spectrum of laboratory variables was measured, which included metabolic syndrome-related indices^9^; diabetes-related indices^10^; uric acids^11^; nutritional indices, including fatty acids,^12^ vitamins, minerals,^9^ alkaloids,^13^ carotenoids,^14^ flavonoids,^5^ sulfur-containing amino acids, and trimethylamine-N-oxide^15^; inflammatory cytokines^16^; indexes of oxidative stress; adipocytes^8^; sexual hormones; liver and renal function-related markers^17^; and routine blood test.

### Multi-omics data

#### Genome-wide genotyping data

Participants with qualified extracted DNA have undergone a genome-wide scan of 750,000 single nucleotide polymorphism (SNP) markers (Illumina Asian Screening Array-750K; Illumina Inc., San Diego, CA, USA), and then genome-wide genotype imputation with the 1,000 Genomes Phase 3 v5 reference panel by Minimac3.^17^

#### Gut microbiome

The research used the 16S rRNA amplicon of feces to sequence the gut microbiota. The V3-V4 hypervariable region of the 16S rRNA gene was amplified and sequenced on Illumina MiSeq System (Illumina Inc.). The 16S rRNA gene is a bacterial ribosomal gene and a part of the 30S subunit, which is used in the identification, characterization, and classification of various bacteria (over 70 gut microbial genera annotated in the research) and microbiota diversity.^18^ Feces were also obtained from participants for metagenomically shotgun sequenced into a library, each also multiplexed by the Illumina HiSeq machine using a 150-bp paired-end read protocol. Metagenomics can be used to study intestinal microbiome diversity and taxonomy. Moreover, functional metagenomics can identify microbial pathways, antibiotic resistance genes, and novel functional genes. More than 160 species and 440 microbial pathways were annotated in the study.^12^ Based on the internal transcribed spacer 2 (ITS2) sequences by thermocycler PCR system (GeneAmp 9700; ABI Scientific Inc., Sterling, VA, USA), a taxonomic profile of the gut mycobiome was determined to investigate gut fungal diversity and composition. There was a total of 204 gut fungal genera identified.^19^

#### Untargeted serum and fecal proteomics

Serum and fecal samples were analyzed using mass spectrometry-based proteomics techniques: Sequential Windowed Acquisition of all THeoretical fragment ion mass spectra^20^ and parallel accumulation-serial fragmentation combined with a data-independent acquisition. Our data library contained 326 unique human protein groups in serum: 1,253 human protein and 83,683 microbial proteins in feces.

#### Targeted metabolomics profiles

Fecal and fasting serum concentrations of metabolites, covering a biologically relevant panel of amino acids, benzenoids, bile acids, carbohydrates, carnitines, fatty acids, indoles, nucleosides, organic acids, organooxygen compounds, phenylpropanoic acids, pyridines, and other metabolites, have been measured using ultrahigh-performance liquid chromatography-tandem mass spectrometry (UPLC-MS/MS) system (ACQUITY UPLC-Xevo TQ-S; Waters Corp., Milford, MA, USA). The Q300 Kit provided by Metabo-Profile Corp. (Shanghai, China), coving up to 310 metabolites and 12 biochemical classes, was used for targeted metabolomics profiling. Approximately 200 metabolites have been quantified in this population.^21^

## RESULTS

The project has obtained more than 90 publications from the research database. It provided data that have been influential in establishing the role of nutrition in metabolic diseases with multi-omics. Below we summarize some of our key findings.

### Nutrition and metabolic health

In the Chinese middle-aged and elderly population, elevated dietary intakes of fruit and vegetables,^22^ betaine,^7^ flavonoids,^23^ serum choline, betaine,^13^ uric acid,^11^ and erythrocyte n-3 polyunsaturated fatty acids (PUFAs)^24^ were associated with better body composition. Erythrocyte membrane de novo lipogenesis-fatty acids^6^ and urinary sodium-potassium ratio^25^ might contribute to worse body composition. Using this cohort study, we found the serum carotenoid levels^14^ and erythrocyte membrane n-3 PUFAs^26^ were inversely associated with risk of nonalcoholic fatty liver disease, while serum retinol-binding protein 4 levels^8^ was positively associated with risk of nonalcoholic fatty liver disease. Our findings suggested that dietary and serum carotenoid levels,^27^ serum isoflavones,^5^ marine-derived erythrocyte n-3 PUFAs,^10^ serum and urinary Mg,^9^ and urinary equol^28^ were potentially cardioprotective and that higher dietary red meat intake,^20^ serum trimethylamine-N-oxide,^15^ erythrocyte gamma-linolenic acid,^29^ and urinary Na and Na/K^30^ were associated with an increased risk of cardiometabolic diseases in middle-aged and older adults.

### Possible pathways with multi-omics techniques

The study is ready to explore pathways related to the association between nutrition and human metabolic diseases. Specimens of most cohort members will be collected from the baseline to the 4^th^ follow-up. We have begun to obtain comprehensive biological changes using multi-omics techniques and found that gut microbiota was a crucial factor in inflammation,^16^ osteoporosis,^4^ diabetes mellitus,^31^ and other complex human diseases.^17^ Furthermore, gut microbiota was found to be a mediator of the associations of erythrocyte n-6 PUFAs^18^ and dietary fruit and vegetable intake^32^ with incident type 2 diabetes (T2D). Our cohort also provided new insights into the interaction of the dairy-gut microbiota^21^; the association of dietary diversity with gut microbiome and fecal metabolites^3^; the association of chronic insomnia with gut microbiota and bile acids^2^; and the association of gut microbiota with acylcarnitine metabolite and equol,^33^ and the relationships of these associations with cardiometabolic health. Our data also described the profile of the gut antibiotic resistome and supported its close relationship with T2D progression.^34^ Our findings underscored the potential role of the gut microbiome in linking genetic variation in CD36, n-3 PUFAs, and lipids, revealing new directions for explaining gene-diet interactions for cardiometabolic health.^12^ We depicted the sociodemographic and dietary determinants of human gut mycobiome in middle-aged and elderly individuals and further revealed that the gut mycobiome might be closely associated with the host metabolic health through regulating gut bacterial functions and metabolites.^19^ In addition, higher red meat intake might interact synergistically with proteomic biomarkers to exaggerate T2D risk.^20^

## DISCUSSION

In 2008, we launched the GNHS project, a cohort designed to provide evidence for the determinants of metabolic diseases in nutritional aspects, as well as other environmental and genetic factors, and explore possible biomarkers and mechanisms with multi-omics integration. The main feature of the GNHS project is facilitating new research by providing multi-omics database containing more than 14 years of follow-up covering a variety of diseases.

As shown in Table 1, compared to the CCFH participants at baseline, the original GNHS participants had less physical activity and more energy intake; were more likely to be higher educated and smokers; and had a lower proportion of calcium supplement users and multivitamin users, possibly owing to a higher proportion of women and older age in CCFH. There were relatively few opportunities for women and older people to receive higher education. On the other hand, women tended to pay more attention to their health status than men. The differences in baseline characteristics between women and men in the GNHS were also largely explained by the fact that women were more concerned about their own health than men.

In this community-based prospective cohort of Chinese population, our findings suggested that n-6 PUFAs and the gut microbiome co-changed during the development of T2D risk, which highlighted a novel mechanism by which fatty acids or the gut microbiome may influence the risk of T2D.^18^ Furthermore, our finding that gut antibiotic-resistant bacteria were broadly associated with fecal metabolites might reflect host-microbe metabolic adaptation.^34^ Bacteria can vertically develop resistance to multiple antibiotics by mutating central housekeeping genes that affect their metabolism.^35^ A study on *Escherichia coli* showed that the acquisition of antibiotic resistance is accompanied by metabolic networks that are specifically reconstituted to circumvent metabolic costs.^36^ Taken together, these findings provide a potential explanation for the mechanism behind the associations observed in this cohort study.

The study has several advantages. First, the research benefits from deep phenotyping, including multiple state-of-art omics (genome-wide genotyping, proteomics, gut microbiome, and metabolomics profiles), instrument examination (MRI, DXA, ultrasound, and 14-d real-time continuous glucose monitoring), a variety of cytokines and nutrients, and biochemical tests, which makes our cohort one of the most profound studies on multi-omics and diseases in the world, since most omics studies are case-control. Second, GNHS has the advantage of having relatively large sample sizes for multi-omics and MRI-related cohort studies. To the best of our knowledge, it has multi-omics and MRI data from thousands of individuals, which is rarely seen in other population-based cohorts. Third, with more than 14 years of face-to-face follow-ups and direct examinations, the quality of data collected in this community based-cohort was relatively high.

However, rare exposures and outcomes cannot be studied in the GNHS program, since a sample size of several thousand participants is limited for exploring the associations between rare exposures and outcomes. Furthermore, the GNHS project recruited participants only in Guangzhou, China and might need to coordinate and link data from multiple cohorts in different areas to achieve broader sample representativeness. In addition, this study was limited by the lack of random sampling due to the need for long-term follow-up.

### Conclusion

The cohort will provide data that will be influential in establishing the role of nutrition in metabolic diseases with multi-omics. The findings of this study will improve our understanding of possible mechanisms in metabolic diseases and provide useful information for the establishment of nutrition prevention strategies for metabolic diseases.

## SUPPLEMENTARY MATERIAL

eMaterial 1. Supplementary methods

eTable 1. Baseline characteristics of the study participants who were in the 2^nd^ follow-up/lost to follow-up^a^ (*n* = 5,118)

eTable 2. Data and biospecimen collections in the GNHS
