# Supplementary material for: Cohort Profile: Guangzhou Nutrition and Health Study (GNHS): A Population-based Multi-omics Study
Source: J Epidemiol. 2024 Jun 5;34(6):301–6. doi: 10.2188/jea.JE20230108 (PMC11078596; doi:10.2188/jea.JE20230108)
Supplement: Supplementary file 1 [file je-34-301-s001.pdf]

eMaterial 1. Supplementary methods

The exclusion criteria for the original Guangzhou Nutrition and Health Study (GNHS)

The exclusion criteria were 1) having a history of hospital-confirmed diabetes, failure(s) of heart, liver, or kidney, cancer, cardiovascular events, metabolic bone diseases, glucocorticoid use (over 3 months), or sexual hormone use (over 6 months), spine or hip fractures; 2) on special diets due to diseases or weight control; 3) mental and physical disability; 4) likely to move to other cities within 5 years; 5) refusing to attend any one item of the study or sample collection.

The exclusion criteria for the controls of a case-control study of hip fractures (CCFH)

Participants who met any of the following criteria were excluded: 1) high-energy fractures or pathological fractures; 2) dietary habits that had changed significantly in the past 5 years; 3) major chronic diseases; 4) medications that currently applied such as thiazine, exogenous estrogens or corticosteroids; 5) poor sight; 6) fracture history. Participants signed an informed consent form for each sub-cohort before enrolment.

Follow-up procedure

The participants were tracked in the 1st follow-up using a telephone invitation (1st follow-up: 2011–2017; N=3,442). A total of 2,895 participants completed the health interview of the second follow-up (2014–2020). The 3rd follow-up began in 2017 and has been ongoing, and 2,243 participants have been revisited so far. About 1,500 participants responded in 2020–now at the 4th follow-up. Now, we have completed 3 waves. The 3rd and 4th follow-ups have

started but have yet to end. Suppose a person is unable to read or recall information, emigrations, dies or unwilling to continue the research. In that case, participation in the study is ended.

Health and well-being measurement

The data collected in this part included general well-being, quality of life, psychological health, and cognitive function using the Self-Rating Anxiety Scale (SAS), Simplified Geriatric Depression Scale (GDS), Mini-Mental State Examinations (MMSE), and Addenbrooke's cognitive examination (ACE), sleeping problems using Pittsburgh Sleep Quality Index (PSQI), family histories, medical histories and medications for major chronic diseases, menstruation and reproductive history (women only), falls, and causes of death from the province-based registration system.

eTable 1. Baseline characteristics of the study participants who were in the 2nd follow-up/lost to follow-up [a] (*n*=5,118)

	Overall	Followed up	Lost to follow-up	*P*-value
n (%)	5,118	2,895 (56.6)	2,223 (43.4)	
	Median (IQR) or *n* (%)			
**Age, years**	59.0 (55.0, 66.9)	57.0 (54.0, 62.0)	65.0 (57.0, 72.0)	**<0.001**
**Women**	3,550 (69.4)	1,989 (68.7)	1,561 (70.2)	0.200
**BMI, kg/m^2**	23.2 (21.3–25.2)	23.1 (21.2–25.2)	23.3 (21.4–25.2)	0.190
**Education**				**<0.001**
Junior high school or below	1,732 (33.8)	771 (26.6)	961 (43.2)	
Secondary high school	2,130 (41.6)	1,317 (45.5)	813 (36.6)	
College degree or above	1,198 (23.4)	768 (26.5)	430 (19.3)	
**Household income, Yuan/month/person**				**<0.001**
≤500	101 (2.0)	45 (2.0)	56 (1.9)	
501–2,000	1,192 (23.3)	461 (20.7)	731 (25.3)	
2,001–3,000	2,047 (40.0)	732 (32.9)	1,315 (45.4)	
>3,000	1,475 (28.8)	697 (31.4)	778 (26.9)	
**Marital status**				**<0.001**
Married or living together	4,413 (86.2)	2,617 (90.4)	1,796 (80.9)	
Divorce/separation/Widowed	639 (12.5)	240 (8.3)	399 (17.9)	
Unmarried/unclassified	66 (1.3)	38 (1.3)	28 (1.3)	
**Smokers[b]**	809 (15.8)	446 (15.4)	363 (16.3)	**<0.001**
**Alcohol drinkers[c]**	357 (7.0)	201 (6.9)	156 (7.0)	**<0.001**
**Tea drinkers[d]**	2,659 (52.0)	1,519 (52.5)	1,140 (51.3)	0.200
**Calcium supplement users**	1,716 (33.5)	915 (31.6)	801 (36.0)	**0.001**
**Multivitamin supplement users**	1,162 (22.7)	639 (22.1)	523 (23.5)	0.057
**Physical activity, MET-h/d[e]**	35.8 (30.2, 48.2)	35.9 (30.6, 51.2)	35.8 (29.4, 46.5)	**<0.001**
**Energy intake, kcal/d**	1,644.1 (1,366.0–2,007.8)	1,693.1 (1,425.6–2,064.3)	1,580.3 (1,312.3–1,912.3)	**<0.001**

BMI, body mass index; IQR, Interquartile range; MET, metabolic equivalent.

[a] Continuous variable were described as medians (IQR, interquartile range) in non-normal distribution, assessed by Wilcoxon tests; categorical variables were described with numbers (%), assessed through chi-square tests.

[b] Smokers were defined as having smoked at least 1 cigarette every day for at least 6 consecutive months.

[c] Alcohol drinkers were defined as taking an alcoholic drink at least once per week for at least 6 consecutive months at any time.

[d] Tea drinkers were defined as taking at least 1 cup of tea per week in the previous 6 months.

[e] Physical activity included daily household chores, walking, standing, stair-climbing, bike-riding, hard physical labor, moderate physical labor, and mild physical labor, assessed by metabolic equivalent (MET) hours per day.

eTable 2. Data and biospecimen collections in the GNHS

Data and specimens	Base-line	Follow-ups 1st	2nd	3rd	4th
**General information and lifestyle**					
Socio-demographic characteristics	✓	✓	✓	✓	✓
Employment-related factors	✓	✓	✓		
Social support and participation	✓	✓	✓	✓	
Behaviours (smoking, passive smoking, alcohol drinking, tea drinking)	✓	✓	✓	✓	✓
Physical activities (19-item questionnaire)	✓	✓	✓	✓	✓
Dietary assessments					
79-item FFQ interviews	✓	✓		✓	
Changes in eating habits	✓	✓	✓	✓	
Use of supplements	✓	✓	✓	✓	✓
7-day image-based food diary					✓
**Health and well-being**					
General well-being		✓	✓	✓	✓
Psychological health					
Self-Rating Anxiety Scale (SAS)	✓				✓
Simplified Geriatric Depression Scale (GDS)					✓
Sleep quality (Pittsburgh Sleep Quality Index, PSQI)				✓	✓
Cognitive function (Mini-Mental State Examinations, MMSE)				✓	✓
Cognitive function (Addenbrooke's cognitive examination, ACE)					✓
Medical history for major chronic diseases	✓	✓	✓	✓	✓
Menstruation and reproductive history (women only)	✓	✓	✓	✓	✓
Family history of diseases	✓	✓	✓		
Medications	✓	✓	✓	✓	✓
Accidents (falling)	✓	✓	✓	✓	
Fecal Bristol scale			✓	✓	✓
Cause of death from the registration system					✓
**Physical examinations**					
Anthropometric measurements	✓	✓	✓	✓	✓
Blood pressure tests	✓	✓	✓	✓	✓
Handgrip strength			✓	✓	✓
Muscle function			✓	✓	✓
Bracelet motion monitoring					✓
**Instrumental examinations**					
DXA scanning					
Whole body (BMD, BMC, fat and lean mass)		✓	✓	✓	✓
Spine L1-L4 (BMD, BMC)		✓	✓	✓	✓
Left hip (BMD, BMC, geometry information)		✓	✓	✓	✓

eTable 2 (continued)

Data and specimens	Baseline	Follow-ups			
		1st	2nd	3rd	4th
Ultrasonography evaluations					
Carotid artery (IMT, plaque, stiffness, etc.)	✓	✓	✓	✓	✓
Fatty liver		✓	✓	✓	✓
Vascular endothelial function evaluation				✓	✓
Cardiopulmonary exercise testing				✓	✓
Magnetic resonance imaging					
Upper-abdomen					✓
Brain					✓
14-d Real-time continuous glucose monitoring tests (With 3-type standard breakfast, 7-d image-based food diary, and 14-d bracelet motion monitoring)					✓
**Biospecimen collection**					
Fasting blood	✓	✓	✓	✓	✓
Early morning first-void urine		✓	✓	✓	✓
Feces			✓	✓	✓
Saliva					✓
**Laboratory tests**					
Metabolic syndrome-related indices	✓	✓	✓	✓	✓
Diabetes-related indices	✓	✓	✓	✓	✓
Uric acid	✓	✓	✓	✓	✓
Nutritional indices (fatty acids, vitamins, minerals, alkaloids, carotenoids, flavonoids, sulfur-containing amino acids and TMAO)	✓	✓	✓		
Inflammatory cytokines	✓				
Index of oxidative stress	✓			✓	✓
Adipocytes		✓			
Sexual hormones		✓			
Liver and renal function-related markers		✓	✓	✓	✓
Routine blood test					✓
**Multi-omics data**					
Genotyping data (Illumina ASA-750K arrays)	✓				
Gut microbiota					
16S rRNA			✓	✓	
Metagenome			✓	✓	OG
ITS2			✓	✓	
Untargeted serum proteomics	✓		✓	✓	
Untargeted fecal proteomics			✓	✓	
Targeted fecal metabolomics			✓		
Targeted serum metabolomics	OG		✓	OG	OG

ASA, Asian Screening Array; BMC, bone mineral content; BMD, bone mineral density; DXA, dual-energy X-ray absorptiometry; FFQ, food frequency questionnaire; IMT, intima-media thickness; ITS2, internal transcribed

spacer 2; OG, on-going; rRNA, ribosomal RNA; Spine L1-L4, lumbar spine segment 1-4; TMAO, Trimethylamine-N-oxide.
